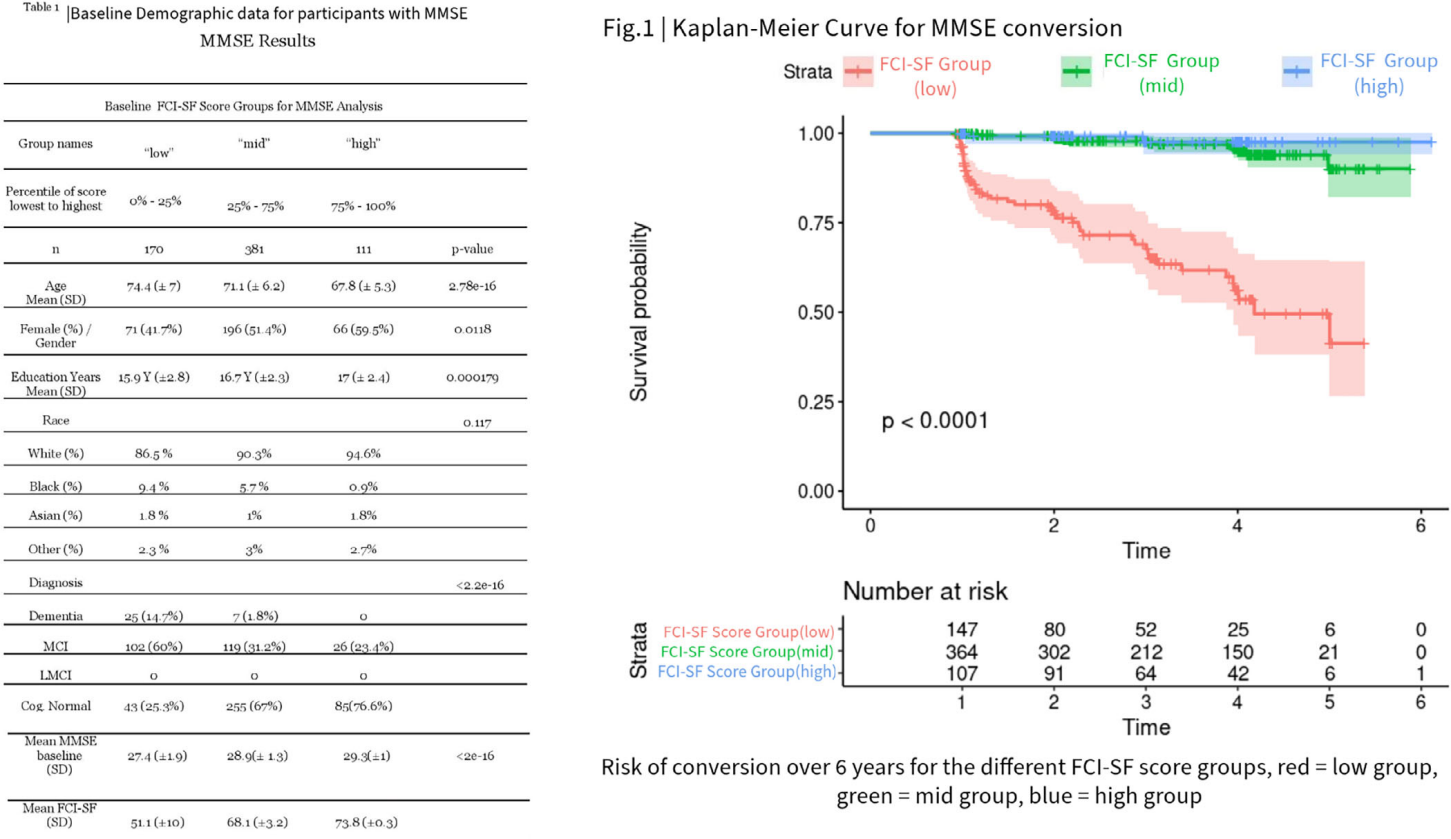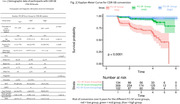# The FCI‐SF predicts Alzheimer’s disease related cognitive changes

**DOI:** 10.1002/alz.093210

**Published:** 2025-01-09

**Authors:** Ella Zak, Thomas Hugentobler Schlickmann, Eduardo R. Zimmer, Marco De Bastiani, Andrei Bieger, Wyllians Vendramini Borelli

**Affiliations:** ^1^ University of applied science Hamm‐Lippstadt, Hamm, North Rhine‐Westphalia Germany; ^2^ Universidade Federal do Rio Grande do Sul, Porto Alegre, Rio Grande do Sul Brazil; ^3^ Federal University of Rio Grande do Sul (UFRGS), Porto Alegre, RS Brazil; ^4^ Universidade Federal do Rio Grande do Sul, Porto Alegre Brazil; ^5^ Brain Institute of Rio Grande Do Sul, PUCRS, Porto Alegre, RS Brazil; ^6^ McGill Centre for Studies in Aging, Montreal, QC Canada; ^7^ Memory Center, Hospital Moinhos de Vento, Porto Alegre, RS Brazil

## Abstract

**Background:**

Recognizing Alzheimer’s disease (AD) early is essential for enhancing patient outcomes. Therefore this research focused on exploring indicators of AD by investigating the role of declining financial capacity (FC) in predicting objetive cognitive impairment (OCI).

**Method:**

We analyzed participants without OCI in Mini‐mental state examination (MMSE, Table 1) or Clinical Dementia Rating‐Sum of Boxes (CDR‐SB, Table 2) scores from the ADNI database. They had a prior completed Financial Capacity Instrument‐Short Form (FCI‐SF). Cox proportional hazard models were used to evaluate how different FCI‐SF scores influence the risk of transitioning to MMSE and CDR‐SB scores that indicate OCI and the timeframe involved. The analyses used the survminer and survival packages in R and corrected for age, gender, diagnose, race, education as covariates (p<0.05).

**Result:**

Participant mean age (SD) for MMSE analysis was 71.3 (±6.6) years (50.8% female). The low FCI‐SF score group (HR = 25.67, 95% CI = 6.232‐105.737) was more likely to convert under the cutoff score for MMSE in the future compared to the high group. Compared to the low group, the high (HR = 0.0389, 95% CI = 0.00945‐0.1605) and mid group (HR = 0.0797, 95% CI = 0.0438‐0.1449) exhibited a lower conversion likelihood.

Mean age for CDR‐SB analysis was 71.1 (±6.4) years (51.8% female). The low group had an increased conversion risk (HR = 9.8128, 95% CI = 3.5171‐27.378) compared to the high group. The high (HR = 0.1019, 95% CI = 0.0365‐0.2843) along with the mid group (HR = 0.1993, 95% CI = 0.1207‐0.3289) had a decreased likelihood to convert compared to the low group.

Converters from the MMSE analysis had a mean (SD) examination time of 2.1 (±1.2) years prior to the conversion. CDR‐SB converters had mean (SD) of 2.2 (±1.3) years. Individuals with higher education had lower conversion likelihood.

**Conclusion:**

A low FCI‐SF score can help predict future MMSE and CDR‐SB outcomes. According to Cox models, individuals with lower FCI‐SF scores have an increased risk of transitioning to MMSE and CDR‐SB scores that indicate OCI.